# Linking cross-species hemodynamics with neuronal activity in prefrontal cortex during abstract sequence viewing

**DOI:** 10.64898/2026.10.23.761183

**Published:** 2026-10-27

**Authors:** Katherine E Conen, Brent Stewart, M Vanessa Rivera Núñez, Hannah Hyde, Nadira Yusif Rodriguez, Aarit Ahuja, Debaleena Basu, Theresa McKim, Theresa M Desrochers

## Abstract

The ability to identify and predict abstract sequential patterns enables a wide range of complex cognition and behavior, but the mechanisms mediating it remain poorly understood. Lateral prefrontal cortex (LPFC) has been implicated in abstract sequence tracking in both humans ^1–5^ and macaques ^5–9^ , but it is unclear whether underlying processes are shared across species. Furthermore, most existing evidence relies on hemodynamic responses, which can have variable relationships with neuronal activity in a region ^10–16^ . We leverage a combination of human fMRI, monkey fMRI, and monkey electrophysiology to bridge these gaps. We find that humans and non-human primates show functionally homologous hemodynamic activation in LPFC during abstract sequence viewing, and neuronal responses within fMRI-targeted regions in macaques contain information about sensory and abstract variables in parallel. However, neuronal activity does not directly mirror hemodynamic responses, and abstract sequence responses are found both near and far from fMRI peaks. In contrast, distinct dynamics of spiking activity are selectively present near fMRI peaks, suggesting that temporal coordination across neurons may influence hemodynamic activity in prefrontal cortex. Together, these experiments establish a link between human brain activity, macaque fMRI, and single neuron responses, providing a window into the neural processes that mediate abstract sequence detection.

## Full Text Availability

The license terms selected by the author(s) for this preprint version do not permit archiving in PMC. The full text is available from the preprint server.

